# Glutathione S-Transferase T1, O1 and O2 Polymorphisms Are Associated with Survival in Muscle Invasive Bladder Cancer Patients

**DOI:** 10.1371/journal.pone.0074724

**Published:** 2013-09-11

**Authors:** Tatjana I. Djukic, Ana R. Savic-Radojevic, Tatjana D. Pekmezovic, Marija G. Matic, Marija S. Pljesa-Ercegovac, Vesna M. Coric, Tanja M. Radic, Sonja R. Suvakov, Biljana N. Krivic, Dejan P. Dragicevic, Tatjana P. Simic

**Affiliations:** 1 Institute of Medical and Clinical Biochemistry, Faculty of Medicine, University of Belgrade, Belgrade, Serbia; 2 Institute of Epidemiology, Faculty of Medicine, University of Belgrade, Belgrade, Serbia; 3 Clinic of Urology, Clinical Centre of Serbia, Belgrade, Serbia; 4 Clinic of Urology, Clinical Centre of Serbia, Faculty of Medicine, University of Belgrade, Belgrade, Serbia; Nanjing Medical University, China

## Abstract

**Objective:**

To examine the association of six glutathione transferase (GST) gene polymorphisms (GSTT1, GSTP1/rs1695, GSTO1/rs4925, GSTO2/rs156697, GSTM1, GSTA1/rs3957357) with the survival of patients with muscle invasive bladder cancer and the genotype modifying effect on chemotherapy.

**Patients and Methods:**

A total of 105 patients with muscle invasive bladder cancer were included in the study. The follow-up lasted 5 years. The effect of *GSTs* polymorphisms on predicting mortality was analyzed by the Cox proportional hazard models, while Kaplan-Meier analysis was performed to assess differences in survival.

**Results:**

*GSTT1 active*, *GSTO1 Asp140Asp* or *GSTO2 Asp142Asp* genotypes were independent predictors of a higher risk of death among bladder cancer patients (HR = 2.5, *P* = 0.028; HR = 2.9, *P* = 0.022; HR = 3.9, *P* = 0.001; respectively) and significantly influenced the overall survival. There was no association between *GSTP1*, *GSTM1* and *GSTA1* gene variants with overall mortality. Only *GSTO2* polymorphism showed a significant effect on the survival in the subgroup of patients who received chemotherapy (*P* = 0.006).

**Conclusion:**

*GSTT1 active* genotype and *GSTO1 Asp140Asp* and *GSTO2 Asp142Asp* genotypes may have a prognostic/pharmacogenomic role in patients with muscle invasive bladder cancer.

## Introduction

Glutathione transferases (GST) are detoxification enzymes that play a role in the conjugation of endogenous or exogenous xenobiotic toxins to glutathione (GSH), however several GSTs function as GSH peroxidases [1]. The family of cytosolic GSTs has different classes including the Alpha (GSTA), Mu (GSTM), Pi (GSTP), Omega (GSTO) and Theta (GSTT) class [1]. Polymorphic expression of GSTA1, GSTM1 and GSTO1 influences the risk of transitional cell carcinoma (TCC) of urinary bladder [2], [3]. Up-regulated GST activity is a hallmark of a malignant phenotype of TCC and is considered important to maintain a prooxidant-antioxidant balance towards a more reduced state in the course of progression of these tumors [4]. Enzymatic activity of GST proteins might influence the capacity of several drugs, used in the treatment of TCC patients, to evoke tumor cell death. Therefore, it is reasonable to assume that common GST polymorphisms may have a prognostic and/or pharmacogenomic role in TCC patients, especially in the case of muscle invasive tumors.

Both MVAC (methotrexate, vinblastine, doxorubicin and cisplatin) and GC/Cis (gemcitabine and cisplatin) protocols used in the treatment of TCC patients with muscle invasive tumors contain drugs (cisplatin and doxorubicin) shown to be substrates for GSTP [5]. The polymorphism of *GSTP1*, resulting in an amino-acid substitution of Isoleucine by Valine (Ile105Val), significantly influences the enzyme activity and is linked to clinical outcome of patients who received platinum-based chemotherapy [6]. Despite the fact that the biotransformation of cisplatin and doxorubicin results in the formation of glutathione conjugates, which are efficiently extruded from the cell by specific export pumps, the data on the role of GSTP1 in limiting the efficacy of the therapy and affecting the survival [7] of patients with muscle invasive TCC are lacking. In addition to GSTP1, *active GSTT1* genotype might influence the capacity of doxorubicin and cyclophosphamide to produce oxidative DNA damage due to its peroxidase activity [8]. Common deletion polymorphisms of *GSTT1* abolish enzyme activity. Recently, it has been suggested that the polymorphisms in genes encoding omega class members GSTO1-1 and GSTO2-2 might also influence the level of oxidative stress, although the mechanisms of differential protein function of various protein isoforms are less well understood. Specifically, GSTO1 and GSTO2 exhibit dehydroascorbate (DHA) reductase activity in addition to novel thioltransferase, and monomethylarsenate reductase activities [9]. GSTO2-2 has 70-100 times higher DHA reductase (DHAR) activity than GSTO1-1 and is considered to be the most active DHAR in mammalian cells [9]. This DHAR activity of GSTO2 may be critical in the maintenance of ascorbic acid (AA) levels not only in normal, but also in the tumor cells. Very recently, it has been shown that both omega SNPs had highly significant effects on gene expression levels of GSTO2, but not of GSTO1 in brain cells [10]. We hypothesized that GST omega polymorphisms might also result in interindividual differences in response to chemotherapeutic protocols in TCC.

In this study we examined the association of six glutathione transferase (GST) gene polymorphisms (GSTT1, GSTP1/rs1695, GSTO1/rs4925, GSTO2/rs156697, GSTM1 and GSTA1/rs3957357) with 5-yr survival in 105 patients with muscle invasive bladder cancer, as well as the genotype modifying effect on chemotherapy.

### Patients and Methods

We enrolled 200 patients newly diagnosed with TCC from the Clinic of urology, Clinical centre of Serbia, Belgrade. Pathological verification of TCC was performed as a part of routine urological practice, including endoscopic biopsy or surgical resection, followed by the histopathological examination by board-certified pathologists. Patients with muscle invasive tumor (105 patients) were considered from the original study group for this particular research. All the participants provided the written informed consent. The study protocol was approved by the Ethical Committee of the Medical faculty, University of Belgrade, and the research was carried out in compliance with the Declaration of Helsinki.

For the 5-year survival analysis, death endpoints were collected from the Serbian Civil Registration System. The follow-up started with the cancer diagnosis and ended with the death or on the 1^st^ November 2012, whichever came first.

Our patients received neoadjuvant MVAC therapy (methotrexate, vinblastine, doxorubicin, cisplatin) or gemcitabine and cisplatin (GC/Cis) combination. The MVAC regimen was given as follows: methotrexate and vinblastine on day 1, 8 and 15, doxorubicin and cisplatin on day 2. Cycles were repeated every 2 weeks. The GC/Cis combination was administered as follows: gemcitabine on day 1, 8 and 15, cisplatin on day 1. Cycles were repeated every 2 weeks. Clinical, hematological, and biochemical assessments were performed prior to every cycle. The exclusion criteria for chemotherapy were impaired renal function, hemoglobin below 100 mg/L, leukocytes below 3000 cell/ml and platelets below 100000 cell/ml.

### GST Genotyping

Genomic DNA was isolated from the whole blood using the QIAGEN QIAmp kit (Qiagen Inc., Valencia, CA, USA).

*GSTA1 C-69T* polymorphism was determined by polymerase chain reaction–restriction fragment length polymorphism (PCR-RFLP) [11]. Used primers were *GSTA1 C-69T* forward: 5′-TGTTGATTGTTTGCCTGAAATT-3′ and *GSTA1 C-69T* reverse, 5′-GTTAAACGCTGTCACCCGTCCT-3′. Presence of restriction site resulting in two fragments (481bp and 385bp) indicated mutant allele (GSTA1*B/B) and if GSTA1*A/B polymorphism incurred it resulted in one more fragment of 96bp.

*GSTM1* genotyping was performed by multiplex PCR [11]. Used primers were *GSTM1* forward: 5′-GAACTCCCTGAAAAGCTAAAGC-3′ and *GSTM1* reverse: 5′-GTTGGGCTCAAATATACGGTGG-3′. Exon 7 of *CYP1A1* gene was co-amplified and used as an internal control using following primers: *CYP1A1* forward: 5′-GAACTGCCACTTCAGCTGTCT-3′ and *CYP1A1* reverse: 5′-CAGCTGCATTTGGAAGTGCTC-3′. The presence of *GSTM1-*active genotype was detected by the band at 215bp, since the assay does not distinguish heterozygous or homozygous wild type genotypes.

*GSTP1 Ile105Val* polymorphism was analyzed using PCR-RFLP method [11]. Used primers were: *GSTP1 Ile105Val* forward: 5′-ACCCCAGGGCTCTATGGGAA-3′ and *GSTP1 Ile105Val* reverse: 5′-TGAGGGCACAAGAAGCCCCT-3′. Presence of restriction site resulting in two fragments (91bp and 85bp) indicated mutant allele (Val/Val) while if Ile/Val polymorphism incurred it resulted in one more fragment of 176bp.

*GSTT1* genotyping was performed by multiplex PCR [11]. Used primers were *GSTT1*-forward: 5′-TTCCTTACTGGTCCTCACATCTC-3′ and *GSTT1*-reverse: 5′-TCACGGGATCATGGCCAGCA-3′. The assay does not distinguish between heterozygous or homozygous wild type genotypes, therefore the presence of 480bp bands was indicative for *GSTT1-*active genotype.

*GSTO1 Ala140Asp* polymorphism was determined by PCR-RFLP method by Marahatta *et al.* [12]. The primers used were *GSTO1 Ala140Asp* forward: 5′-GAA CTT GAT GCA CCC TTG GT-3′ and *GSTO1 Ala140Asp* reverse: 5′-TGA TAG CTA GGA GAA ATA ATT AC-3′. The presence of restriction site resulting in two fragments (186 and 68 bp) indicated *AlaAla* wild type homozygote, and if *AlaAsp* heterozygote incurred, it resulted in one more fragment of 254 bp.

*GSTO2 Asn142Asp* polymorphism was determined by PCR-RFLP method by Marahatta *et al.* [12]. The primers used were *GSTO2 Asn142Asp* forward: 5′-AGG CAG AAC AGG AAC TGG AA-3′ and *GSTO2 Asn142Asp* reverse: 5′-GAG GGA CCC CTT TTT GTA CC-3′. The presence of restriction site resulting in two fragments (122 and 63 bp) indicated *AspAsp* homozygote of polymorphic sequence, while if *AsnAsp* heterozygote incurred, it resulted in one more fragment of 185 bp.

### Statistical analysis

Statistical analysis was performed using the Statistical Package for the Social Sciences (SPSS, version 15.0; SPSS Inc, Chicago, Illinois, USA). Survival analysis was performed separately in the total cohort and in the subgroup of patients who received chemotherapy. The Kaplan-Meier method was used to estimate the cumulative survival probability. The long-rank test was performed for the assessment of differences in survival according to the different categories of variables.

The predictive value of different GST genotypes in overall mortality was assessed by the Cox proportional hazard regression models, adjusted by the confounding factors in two models. In Model 1 the adjustments were made for age and gender. Model 2 included the covariates in Model 1 plus an additional adjustment for the grade. The associations are presented as hazard ratios (HR) with their corresponding 95% confidence intervals (95% CI).

## Results

The patients’ characteristics are presented in Table 1. The mean follow-up was 38.2±23.1 months (ranging from 1 to 66 months). Genotyping was attempted in 105 patients and in 101 of patients genotyping was successful for all genotypes tested. Of 101 patients, 62 died (61.4%) from urinary bladder cancer and 12 patients (11.9%) were lost during the follow-up.

**Table 1:** Patient characteristics at study entry.

Characteristic	No. of patients	%
**Total No. of patients**	101	
**Age (mean ± SD)**	64.04±9.34	
**Gender**		
Male	77	76.2
Female	24	23.8
**Grade**		
G1	5	5
G2	36	35.6
G3	60	59.4
**Stage**		
2	42	41.6
3	37	36.6
4	22	21.8
**Chemotherapy**		
Yes	51	50.4
No	50	49.5

The genotype distribution of *GSTT1, GSTP1, GSTO1, GSTO2, GSTM1* and *GSTA1* is presented in Table 2. The frequency of *GSTT1, GSTP1, GSTM1* and *GSTA1* genotypes in TCC patients corresponds to that already reported in TCC patient cohort (Matic M, accepted for publication in Urologic Oncology). The frequency of GST omega class gene variants is in the accordance with the data of Lesseur et al [13].

**Table 2:** Glutathione S-transferase (GST) genotype distribution.

Gene	rs	Genotype	Distribution (%)
*GSTT1*		*GSTT1 active*	76
		*GSTT1 null*	24
*GSTP1*	rs1695	*GSTP1 Ile105Ile/Ile105Val*	88
		*GSTP1 Va105Val*	12
*GSTO1*	rs4925	*GSTO1 Ala140Ala/Ala140Asp*	92.1
		*GSTO1 Asp140Asp*	7.9
*GSTO2*	rs156697	*GSTO2 Asn142Asn/Asn142Asp*	90.1
		*GSTO2 Asp142Asp*	9.9
*GSTM1*		*GSTM1 active*	40.6
		*GSTM1 null*	59.4
*GSTA1*	rs3957357	*GSTA1 AA/AB*	87.1
		*GSTA1 BB*	12.9

**Figure 1:**
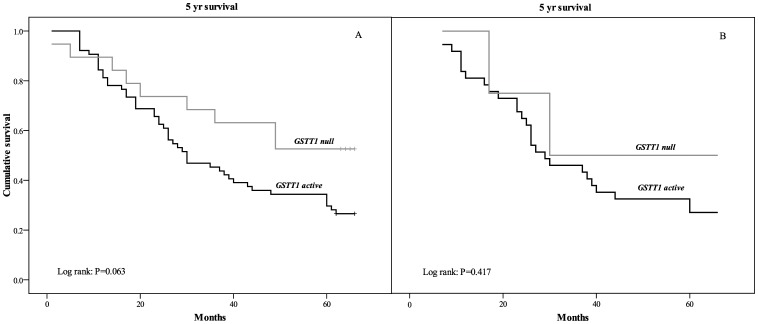
Survival analysis Kaplan–Meier curves according to *GSTT1* polymorphism for overall mortality (A), as well as mortality of TCC patients on chemotherapy (B).

Table 3 summarizes the association of six GSTs polymorphisms and overall mortality in TCC cohort. The presence of the *GSTT1 active* genotype was an independent predictor of a higher risk of overall mortality among TCC patients (HR = 2.5, 95% CI: 1.1–5.5; *P* = 0.028; Table 3). The Kaplan-Meier survival analysis in the whole group of TCC patients demonstrated shorter mean overall survival after the diagnosis of TCC in patients with *GSTT1 active* alleles in comparison with carriers of double deleted *GSTT1* alleles (36.9±2.8 vs. 46.4±5.4 months, respectively; *P* = 0.063; Fig.1a). Patient survival with respect to *GSTT1* was not affected by chemotherapy treatment (Fig 1b).

**Table 3:** *GSTT1, GSTP1, GSTO1, GSTO2, GSTM1* and *GSTA1* polymorphisms as the predictors for overall mortality among 101 patients with muscle invasive TCC after 5 yrs of follow-up by the Cox proportional hazards regression.

Model 1^a^		Model 2^b^	
HR (95% CI)	*P*-value	HR (95% CI)	*P*-value
**Risk of overall mortality comparing ** ***GSTT1 active to GSTT1 null*** ** genotype**			
2.032 (0.989–4.173)	0.054	2.471 (1.101–5.545)	0.028
**Risk of overall mortality comparing ** GSTP1*Ile ** carriers to ** GSTP1*Val ** homozygotes**			
2.102 (0.128–5.336)	0.118	2.071 (0.723–5.935)	0.175
**Risk of overall mortality comparing ** GSTO1*Asp ** homozygotes to ** GSTO1*Ala ** carriers**			
1.962 (0.812–4.744)	0.134	2.941 (1.164–7.430)	0.022
**Risk of overall mortality comparing ** GSTO2*Asp ** homozygotes to ** GST02*Asn ** carriers**			
2.870 (1.355–6.076)	0.006	3.967 (1.760–8.939)	0.001
**Risk of overall mortality comparing ** ***GSTM1 null*** ** genotype to ** ***GSTM1 active***			
1.062 (0.609–1.852)	0.833	1.128 (0.620–2.052)	0.694
**Risk of overall mortality comparing ** GSTA1*B homozygotes ** to ** GSTA1*A ** carriers**			
1.190 (0.671–2.112)	0.552	1.387 (0.615–3.127)	0.430

Abbreviations: CI, Confidence Interval; HR, Hazard Ratio.

a Adjusted for age and gender.

b Adjusted for the covariates in Model 1 plus an additional adjustment for grade.

**Figure 2:**
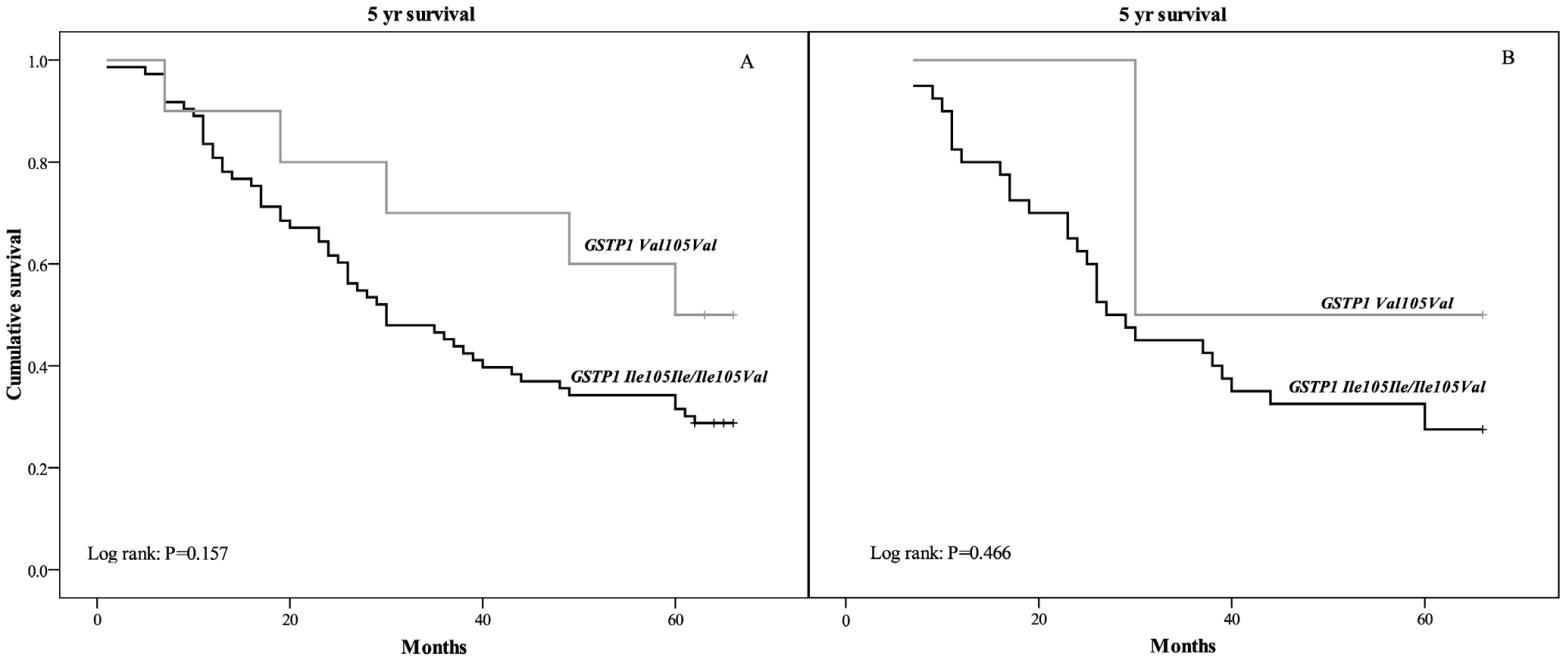
Survival analysis Kaplan–Meier curves according to *GSTP1* polymorphism for overall mortality (A), as well as mortality of TCC patients on chemotherapy (B).

Regarding *GSTP1* polymorphism, *GSTP1 Ile* allele carriers were at a higher risk of overall mortality and had a multivariable adjusted (model 2) HR of 2.1 (95% CI: 0.7–5.9; *P* = 0.175; Table 3) in comparison with GSTP1 *Val* homozygotes. The Kaplan-Meier survival analysis in the whole group of patients demonstrated shorter mean overall survival in patients carriers of at least one *GSTP1 Ile* allele compared to *GSTP1 Val* homozygotes (36.9±2.7 vs. 49.5±6.8 months, respectively; *P* = 0.157; Fig. 2a). Patient survival with respect to *GSTP1 Ile105Val* was not affected by chemotherapy treatment (Fig 2b).

**Figure 3:**
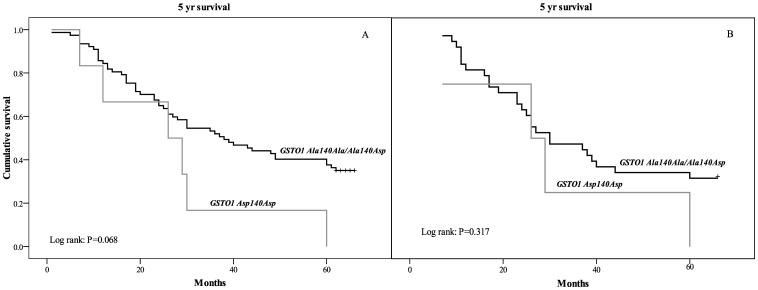
Survival analysis Kaplan–Meier curves according to *GSTO1* polymorphism for overall mortality (A), as well as mortality of TCC patients on chemotherapy (B).

Regarding *GSTO1* polymorphism, the presence of both mutant alleles (*Asp140Asp)* was found in 8 patients and was an independent predictor of a higher risk of overall mortality (HR = 2.9, 95% CI: 1.2–7.4; p = 0.022; Table 3). Although, *GSTO1 Asp140Asp* genotype has a minor frequency, it has a great functional significance since 6 of 8 patients with this genotype died during 5 yrs of follow-up. The Kaplan-Meier survival analysis in the whole group with TCC has shown shorter overall survival after TCC diagnosis in patients carriers of GSTO1 *Asp140Asp* in comparison with carriers of at least one *GSTO1 Ala* allele (27.3±7.6 vs. 40.1±2.6 months, respectively; *P* = 0.068, Fig 3a). Patient survival with respect to *GSTO1* was not affected by chemotherapy treatment (Fig 3b).

**Figure 4:**
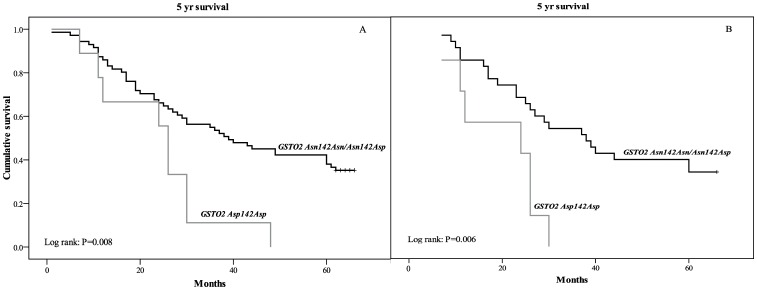
Survival analysis Kaplan–Meier curves according to *GSTO2* polymorphism for overall mortality (A), as well as mortality of TCC patients on chemotherapy (B).

Similarly to *GSTO1*, the presence of both mutant *GSTO2* alleles (*Asp142Asp)* was an independent predictor of a higher risk of overall mortality among TCC patients. This genotype had the most pronounced effect in terms of mortality risk since a multivariable adjusted (model 2) HR of 3.9 (95% CI: 1.8–8.9; *P* = 0.001; Table 3) was observed. Nine of 10 patients with GSTO2 *Asp142Asp* genotype died within 5 yrs of follow-up. The Kaplan-Meier survival analysis in the whole group with muscle invasive TCC demonstrated that the *GSTO2 Asp142Asp* homozygotes had two times shorter mean survival in comparison with patients with at least one *Asn* allele (23.8±4.2 vs. 40.8±2.7 months, respectively; *P* = 0.008, Fig 4a). Survival analysis in a subgroup of patients who received chemotherapy has shown statistically significant shorter survival in patients carriers of both mutant alleles compared to carriers of at least one *GSTO2 Asn* allele (19.4±3.4 months vs. 40.3±3.7 months, respectively; *P* = 0.006, Fig 4b).

There were no robust statistical associations between GSTM1 and GSTA1 gene variants with overall mortality according to the Cox (Table 3) and the Kaplan-Meier analyses (data not shown).

## Discussion

In the present study we have shown that *GSTT1 active* genotype or homozygous mutant *GSTO1 Asp140Asp* and *GSTO2 Asp142Asp* genotypes are associated with worse prognosis and shorter survival in muscle invasive bladder cancer patients. Besides, *GSTP1 Ile* carriers exhibited increased, but insignificant, overall mortality risk.

Homozygous deletion in *GSTM1* gene has been associated with shorter survival after diagnosis in 95 bladder cancer patients as shown by Nørskov et al [14]. In the present study we didn’t find the association between *GSTM1 null* genotype and survival in muscle invasive TCC patients. On the other hand, *GSTT1 active* genotype has been associated with a lower recurrence- and progression-free survival in patients with non muscle-invasive TCC [15]. Similar prognostic significance of *GSTT1* has also been shown in patients with muscle-invasive TCC in this investigation. The risk conferred by *GSTT1 active* allele on mortality might be explained in terms of both antioxidant activity of GSTT1 protein and its potential role in the inactivation of free radicals produced by anticancer drugs. During the growth of TCC, important changes occur in cell redox homeostasis which might affect apoptotic signaling pathways [16]. The oxidant–antioxidant balance in TCC most probably favors the reduced state as the increased levels of reduced glutathione were reported in these tumors. Additionally, up-regulated activities of antioxidant enzymes including GSTT1 have also been observed in TCC [16], [17]. It may be speculated that GSTT1 up-regulation does not occur in TCC patients with *GSTT1 null* genotype. Therefore, better outcome of these patients may be explained by the absent antioxidant GSTT1 activity, which favors more byproducts of oxidative stress in tumor cells and slower tumor progression. Similar prognostic role of *GSTT1 active* genotype has also been found in osteosarcoma [18]. The question arises whether any of the drugs used in the therapy of TCC patients represents a GSTT1 substrate. Diedrich et al. [19] showed that GSTT1 may be considered as a relevant factor for chemotherapy of glioblastomas. Although, there are no data on the role of GSTT1 in the inactivation of drugs in MVAC and GC/Cis protocols, at least two of them (doxorubicin and cisplatin) exert their mechanism of action through reactive oxygen species generation as well as apoptotic pathway activation [20]. In our study further stratification of patients according to chemotherapy treatment did not show significant effect of *GSTT1* polymorphism on survival rate. Therefore, it may be concluded that *GSTT1* genotype has more influence on tumor progression by altering its redox balance then influencing the metabolism of free radicals produced by anticancer drugs.

The role of *GSTP1* polymorphism in chemotherapy resistance has been unambiguously documented in vast majority of both in vivo and in vitro studies [6], [21]. Cisplatin and doxorubicin are proven substrates for GSTP1, with *GSTP1 Ile* as a variant with more affinity [5]. In this study, we did not find the significant association between *GSTP1* polymorphism and chemotherapy response, although patients with *Val/Val* genotype demonstrated longer survival than carriers of at least one *Ile* allele. This beneficial effect of *GSTP1 Val/Val* genotype on survival has also been found in the whole group of TCC patients, including those who did not receive chemotherapy. Although, apoptosis inhibition mediated by GSTP1:JNK interaction is a key mechanism in the progression of TCC [22], there are no data on the differential antiapoptotic activity of various GSTP1 proteins (Ile or Val) regarding JNK binding and apoptosis inhibition. However, the most recent data demonstrate that the polymorphic expression of GSTP1-1 in MCF-7 cells differentially mediate the activation of GSTP-associated Prdx6 peroxidase activity, providing a platform to imply that contingent upon their *GSTP* genotype, individuals will have the significant differences in mounting an antioxidant response [23]. If these results are translated to TCC setting, it may be speculated that *GSTP1 Ile/Ile* would imply a higher antioxidant potential providing the favorable environment for tumor progression and worse prognosis.

The functional role for polymorphisms in both GSTO1 and O2 has started to emerge recently as Allen et al. [10] have shown that *GSTO1 AspAsp* and *GSTO2 AspAsp* had highly significant effect on lower brain gene expression levels of GSTO2, but not of GSTO1. If such kind of regulation of GSTO2 expression also exists in TCC cells, *GSTO1* or *GSTO2* polymorphisms would presumably result in deficient DHAR activity, and lower ascorbic acid level (AA) in tumor. Since, the majority of TCC patients with *GSTO1* or *GSTO2* polymorphisms died, it would imply the anti-tumor role of AA in TCC progression. Given the traditional role of AA as an antioxidant, these results would contrast the postulate that reduced environment favors the growth of TCC. However, it seems that the antitumor effect of AA has a biologically plausible explanation, since AA functions also as a pro-oxidant and may promote apoptosis in tumor cells such as colon carcinoma cell line [24]. Future studies on correlation between *GSTO* genotype and phenotype (GSTO2 protein level and DHAR activity) as well as AA measurements in TCC would be necessary to provide explanation for the fact that TCC patients with mutant *GSTO2 Asp142Asp* genotype had a 3.9-fold increased risk of death in comparison with carriers of at least one *GSTO2 Asn* allele, while those with *GSTO1 Asp140Asp* genotype exhibited 2.9- fold increased risk of death than patients with at least one *Ala* allele. It is important to note that *GSTO2* polymorphism was the only one which showed a significant effect on survival in the subgroup of TCC patients who received chemotherapy. Namely, TCC patients with both *GSTO2 Asp* mutant alleles had two times shorter survival in comparison with carriers of at least one *Asn* allele. This further strengthens our hypothesis on anti-tumor role of AA in TCC. This assumption is in reference with results of Catani et al [24], who showed that AA increases the anti-neoplastic activity of cisplatin thereby increasing the apoptosis of tumor cells. More data on the potential application of ascorbic acid for therapeutic purposes in various tumors have been described in review of Li and Schellhorn [25].

Certain limitations could be considered in our study. Relatively small numbers of the study participants and GST polymorphisms studied might be the sources of potential biases which may influence the study findings. However, we tested effects of six GST polymorphisms on outcome in TCC patients and therefore significantly decreased a chance for publication bias. Namely, positive results of genetic studies analyzing a small number of polymorphisms (n = 1–3) should be evaluated cautiously and considered at a lower level of evidence [26]. Besides, our patients were treated with polychemotherapy and for this reason it is difficult to establish the effect of GSTs polymorphisms on treatment outcome for each particular drug. Nevertheless, this study may offer some essential information that could be the base for future longitudinal research, especially regarding the GST class omega polymorphisms.

Taken together, our data suggest that *GSTT1 active* genotype or homozygous mutant *GSTO1 Asp140Asp* and *GSTO2 Asp142Asp* genotypes are associated with worse prognosis and shorter survival in muscle invasive bladder cancer patients. GSTT1, GSTO1 and GSTO2 as genetic markers may have a prognostic or pharmacogenomic role in patients with muscle invasive bladder cancer.
